# Novel cattail fiber composites: converting waste biomass into reinforcement for composites

**DOI:** 10.1186/s40643-021-00453-8

**Published:** 2021-10-13

**Authors:** Md. Shadhin, Mashiur Rahman, Raghavan Jayaraman, Danny Mann

**Affiliations:** 1 Composite Materials and Structures Research Group and Department of Mechanical Engineering, University of Manitoba, Winnipeg, MB R3T 5V6 Canada; 2 Department of Biosystems Engineering, University of Manitoba, Winnipeg, MB R3T 5V6 Canada

**Keywords:** Cattail fiber, Waste biomass, Non-woven mat, VARTM, Compression molding, Natural fiber composite

## Abstract

Vacuum-assisted resin transfer molding (VARTM), used in manufacturing medium to large-sized composites for transportation industries, requires non-woven mats. While non-woven glass mats used in these applications are optimized for resin impregnation and properties, such optimized mats for natural fibers are not available. In the current research, cattail fibers were extracted from plants (18–30% yield) using alkali retting and non-woven cattail fiber mat was manufactured. The extracted fibers exhibited a normal distribution in diameter (*d*_avg._ = 32.1 µm); the modulus and strength varied inversely with diameter, and their average values were 19.1 GPa and 172.3 MPa, respectively. The cattail fiber composites were manufactured using non-woven mats, Stypol polyester resin, VARTM pressure (101 kPa) and compression molding pressures (260 and 560 kPa) and tested. Out-of-plane permeability changed with the fiber volume fraction (*V*_f_) of the mats, which was influenced by areal density, thickness, and fiber packing in the mat. The cattail fibers reinforced the Stypol resin significantly. The modulus and the strength increased with consolidation pressures due to the increase in *V*_f_, with maximum values of 7.4 GPa and 48 MPa, respectively, demonstrating the utility of cattail fibers from waste biomass as reinforcements.

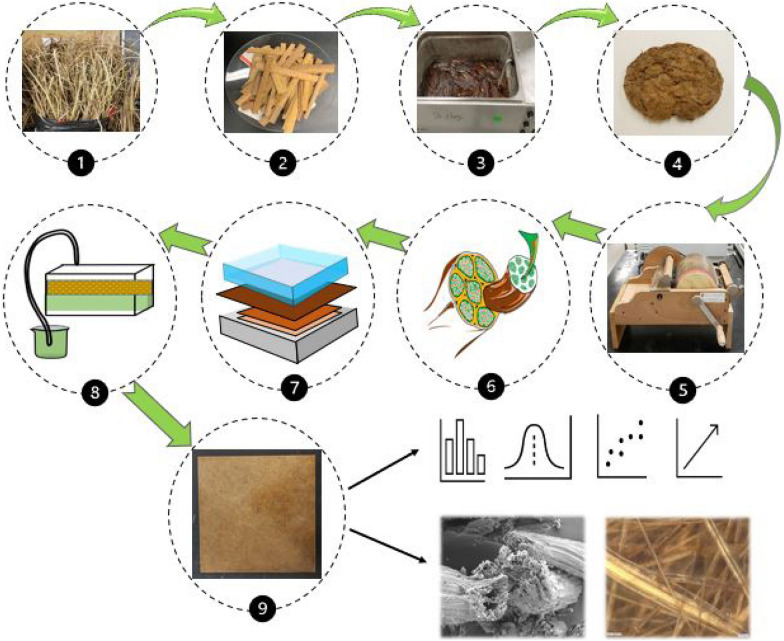

## Introduction

Polymer–matrix composites (PMC) are increasingly used in structural applications. PMC can be categorized as particulate composites, discontinuous/short-fiber composites, and continuous fiber composites. Continuous fiber composites are used in structural applications in the aerospace industry, such as fairings, vertical and horizontal stabilizers, and fuselage, where meeting the desired properties is more important than the cost. However, discontinuous fiber composites are usually used in semi-structural or non-structural applications such as doors, window frames, and automotive interior parts, where cost is the primary consideration (Campbell 2010; Mazumdar 2001).

Natural fiber-reinforced composites (NFRC) are gaining interest due to the renewability of natural fibers over synthetic fibers currently in use. The natural bast fibers (BFs), such as flax, kenaf, jute, hemp, and sisal are increasingly being investigated as environmentally friendly alternatives to glass fibers in engineering applications (Fahimian 2013; Nishino et al. 2003; Karnani et al. 1997; Oksman et al. 2002; Wambua et al. 2003; Wrobel et al. 2012; Yan et al. 2014). The mechanical properties of NFRC rely on the fiber properties, fiber geometry, fiber orientation, and fiber volume fraction (Lau et al. 2018; Ho et al. 2012).

Cattail (*Typha latifolia*) fiber is a waste biomass fiber that is easy to extract, using an alkaline solution, from their raw resources. Cattail fiber has several advantages over BFs, which include lower density (1.26 g/cm^3^), abundant supply without any cost for growing them, and higher fiber yield of about 40–60% (Mortazavi and Moghadam 2009; The Canadian Encyclopedia 2015; Chakma 2018; Rahman et al. 2021).

Unlike BFs that are grown as the main crop, cattails grow naturally in bog and fen, lacustrine marshes, prairie pothole marshes, roadside ditches, riverine marshes, tidal marshes, and are becoming increasingly dominant wetland plants in North America (Shih and Finkelstein 2008). The cellulosic content of cattail fiber is similar to that present in BF (Faruk et al. 2012; Vetayasuporn 2007).

Previously, cattail plants have been investigated for composite applications using whole cattail leaves (Stanescu and Bolcu 2019; Bazwa et al. 2015), decorticated cattail leaves (Wuzella et al. 2011; Mbeche et al. 2020), milled cattail leaf mesh (Kongkaew et al. 2018), and individual fiber without conversion into non-woven mats (Sana et al. 2015). The extraction of textile-grade fiber from the cattail leaves has been demonstrated by Rahman et al. (2021). However, non-woven preforms are required for manufacturing composite using VARTM. Mechanical properties of the manufactured composite part depend on the non-woven mat structure (areal density, fiber volume fraction (*V*_f_) and consolidation of the mat under manufacturing pressure). Shadhin et al. (2021) and Fahimian (2013) have correlated the effect of mat properties and consolidation pressure on composite properties for flax and hemp, respectively. However, such knowledge for cattail fibers is lacking and is required for the adoption of these fibers as reinforcement in composites manufactured using VARTM. Hence, this research is focused on generating this knowledge and evaluating the suitability of cattail fibers for composite applications. Cattail fibers were extracted from the leaves and preformed to obtain non-woven mats. Composites were manufactured using these mats, VARTM and compression molding. Mechanical properties of these composites were measured and evaluated to demonstrate the suitability of these fibers in composite applications.

## Experimental details

### Materials

**Fig. 1:**
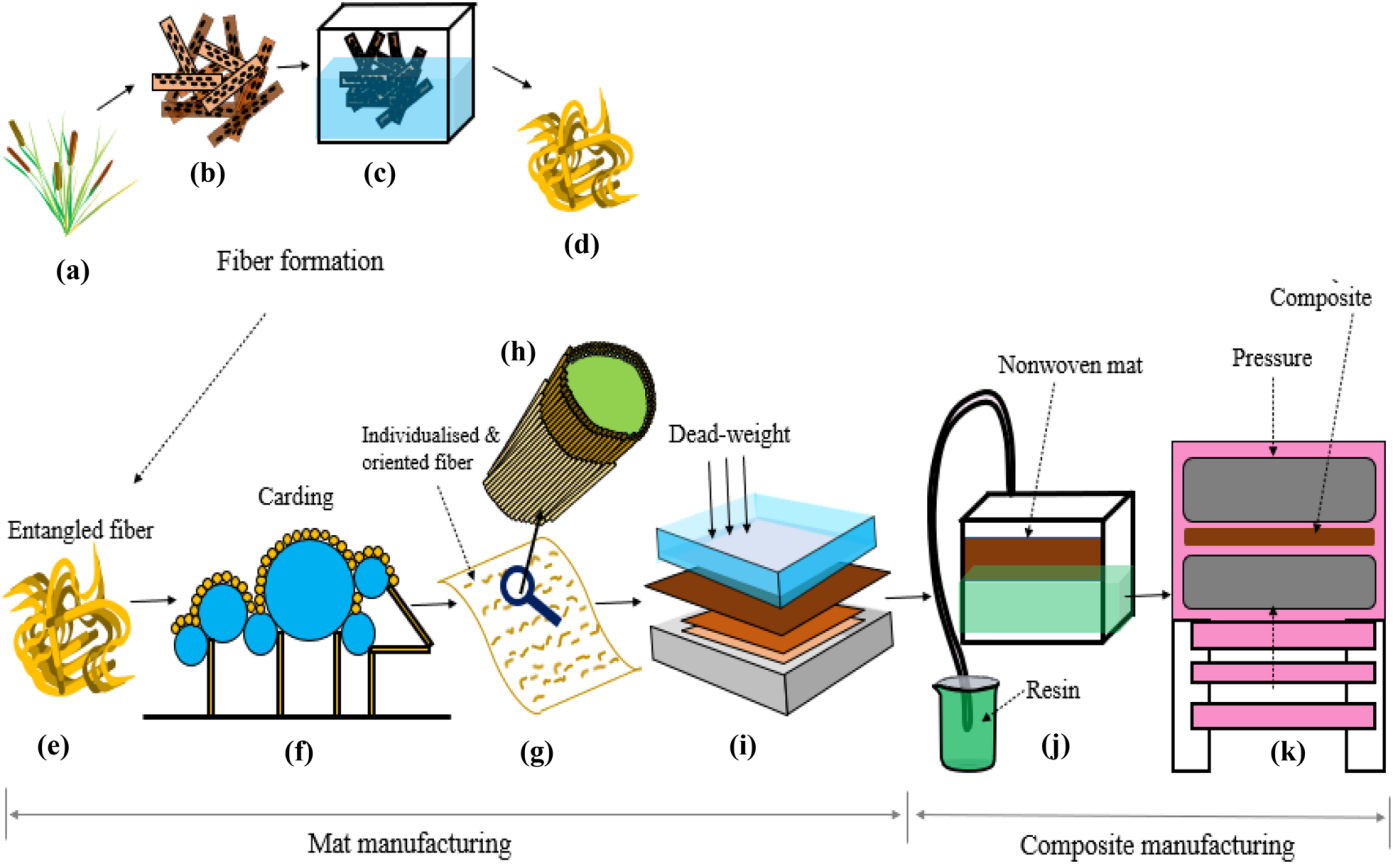
Schematic diagram for manufacturing compression molded composites using fibers from cattail leaves. **a** Green cattail plants. **b** 6–10 cm precut cattail leaves. **c** Alkali retting of cattail (90 °C, 4 h) 5% KOH. **d** Extracted cattail fibers after drying. **e** Entangled fiber bundles fed to carding machine. **f** Disentangling and combing of fibers via carding process. **g** Individualized, parallelized, and oriented fiber bundle. **h** Single cattail fiber. **i** Dead weight applied on fiber layers placed on template. **j** Non-woven mat impregnation by resin in VARTM.**k** Impregnated mat sandwiched between metal plates, release film, and silicon pad (compression molding)

Green cattail plants were collected from the roadside ditches along Provincial Highway 3 near Winnipeg, Canada in early October 2019 (Fig. 1a). Aqueous KOH (Fisher Scientific, Ontario, Canada) was used for fiber extraction while acetic acid (Fisher Scientific, Ontario, Canada) was used for the neutralization of the fiber after extraction. Unsaturated polyester resin (Stypol 8086) was used as the thermoset polymer matrix (Composite Envisions LLC, Wausau, USA). It is a low-viscosity resin, which starts to cross-link with the addition of a curing initiator. The curing initiator chosen for this study was Luperox 224 (2,4-Pentanedione peroxide, Sigma Aldrich, Oakville, Ontario, Canada).

### Extraction of cattail fibers and manufacturing of non-woven mats

The steps involved in the transformation of the cattail leaves into fibers, non-woven mats and composites are shown schematically in Fig. 1a–k.

#### Fiber extraction

The collected cattail leaves were dried at room temperature for 48 h and precut to 6–10 cm in length (Fig. 1b) and weighed. In the beginning, fiber extraction conditions were varied by varying temperature (70, 80, and 90 °C), time (2, 3, and 4 h) and alkaline concentrations (1, 2, 5, and 10%). 90 °C temperature and 4 h treatment in 5% KOH were chosen to be the optimal extraction condition based on the individuality of the extracted fiber (single fiber entity) and flexibility. This assessment procedure was based on a previous study by Rahman et al. (2021). The softness of Typha fiber was evaluated using the AATCC evaluation procedure 5 (AATCC 2010). In this method, fiber bundles were taped to a piece of cardboard and placed into a 50.8 mm^2^ × 50.8 mm^2^ polybag and evaluators graded them for softness in the range of 1–5 (lowest to highest). The individuality of the fibers was determined using the Bioquant software which is connected to a computer, a projection microscope, and a camera (Bioquant, Nashville, USA).

A stock solution of 5% (w/v) KOH was prepared and the required amount (250 g) of cattail leaves was added to it. The temperature of the mixture was controlled using a water bath (capacity: 12–15 L) covered with a lid (Fig. 1c). Once the fibers separated from the digested leaves, they were rinsed in cold distilled water and neutralized in 2% (v/v) acetic acid solution for 30 min, and then were subsequently washed progressively in cold, hot, and cold distilled water and left to dry at room temperature (Fig. 1d). The above procedure was repeated for 30 extraction runs.

#### Manufacturing mat

The extracted cattail fibers (Fig. 1e) were individualized by passing them between spiked rollers of a modified laboratory carding machine (Fig. 1f). While the spiked roller helped to individualize the entangled fibers obtained from extraction, the combing operation, during each pass, helped to orient the individual cattail fibers parallel to one another (Fig. 1g and h). Subsequently, these fibers were used with a customized template to manufacture non-woven mats. The template consisted of a metal platen (21.5 cm × 21.5 cm) covered by a paper board on each side for ease of thickness control while laying up individualized fibers. The fibers were dropped by hand and allowed to deposit into the mold by gravity in order to avoid preferential orientation. Once the fibers were laid, another metal platen with the same dimension was placed on top of the mat and a dead-weight of 3 kg (6 × 0.5 kg) was applied to compress the fiber bed (Fig. 1i).

### Composite manufacturing

The composite was manufactured using a VARTM mold. Stypol 8086 mixed with (2%—w/w) the LUPEROX 224 initiator was degassed and injected into the mat under vacuum. After impregnation under vacuum pressure (~ 101 kPa), the composite was allowed to cure overnight (24 h) at room temperature. Additional mats were cured under various consolidation pressures to study the effect of pressure. The mats impregnated using the VARTM set-up were removed from the mold after resin impregnation, and were compression molded in a hydraulic press under the chosen pressure. The impregnated mat was sandwiched between two release films, which were subsequently sandwiched between two metal plates and two silicone pads and subjected to pressures of 260 and 560 kPa, using a G50 H-24-CLX hydraulic press manufactured by WABASH MPI, IN, USA. The composites were left in the press for 8–10 h to cure at room temperature.

### Fiber characterization

#### Fiber yield

The fiber yield (%) was determined as the ratio of the oven-dried mass of the fibers extracted after chemical treatment to the oven-dried mass of the cattail plants before chemical treatment.

#### Single-fiber tensile testing

The mechanical properties, i.e., tensile strength, modulus of elasticity, and strain at break (%) of cattail fiber were measured using an Instron Tensile Tester (Model# 5965, Sl#VS02075661, Norwood, USA) following the ASTM D3822-14 (ASTM 2020) method. Single cattail fibers were bonded to a paper frame with a rectangular hole in the center. Before tensile testing, the cattail fiber’s diameter was measured using an image analyzer (Bioquant life science—Motic, BA310l, 2010). The length of the fiber inside the rectangular hole of the frame (i.e., 25 mm) acted as the gage length to measure the strain. After clamping the frame, bonded with the fibers between the clamps of the Instron tester, the paper frame was cut at the center so that the tension was applied only on the fiber. Tensile testing was done at a crosshead speed of 20 mm/min, using a 1-kN load cell.

### Mat characterization

#### Thickness, areal density and *V*_f_

The thickness of each non-woven mat was measured using a caliper. For areal density (gsm—gram per square meter) measurement, the weight of the manufactured mat sample for a given area (21.5 cm × 21.5 cm) was recorded. The fiber volume fraction (*V*_f_) in the non-woven mat was determined using Eq. (1):

1 $${\text{Fiber volume fraction}}, \,V_{{\text{f}}} \left( \% \right) = \frac{W}{{A h \rho_{{\text{f}}} }}$$

where *W* is the weight of the cattail mat, *A* is the area, *h* is the mat thickness, and *ρ*_f_ is the density of the reinforcing fiber.

#### Out-of-plane permeability

A Frazier permeability tester (manufactured by Frazier Precision Instrument Co. Inc. Hagerstown, MD, USA) was used in this study to determine the volumetric flow rate through the non-woven cattail mat as per the ASTM D-737-18 (ASTM 2018) test method. The airflow rate through the thickness of a non-woven mat of a known area was adjusted to obtain a prescribed air pressure drop (equivalent to 0.5 in. of water) across the thickness. The out-of-plane permeability (i.e., through-the-thickness), *k*_*z*_, was calculated using Darcy’s law in Eq. (2):

2 $$k_{z} = \frac{Q \eta L }{{A \Delta P}}$$

where *Q* = volumetric flow rate; *η* = viscosity of air = 1.81 × 10^–5^ Pa s; *A* = area of the specimen perpendicular to flow direction = 0.003788 m^2^; ∆*P* = pressure difference, and *L* = length of mat parallel to the flow direction.

### Composite characterization

#### Testing

The mechanical properties of the manufactured composite were determined using an MTS tensile testing machine with 30-kN load cell and extensometer with 50.8 mm gage length, following ASTM D3039-17 (ASTM 2017). All test coupons were stored in the lab atmosphere (45% relative humidity, 22 °C) after preparation until testing. The testing was done at a crosshead speed of 2 mm/min. Five coupons were tested for each consolidation pressure. The tensile modulus of manufactured mat composite was calculated from the slope of the stress–strain curve from the initial linear portion in the strain range of 0.1%.

**Fig. 2:**
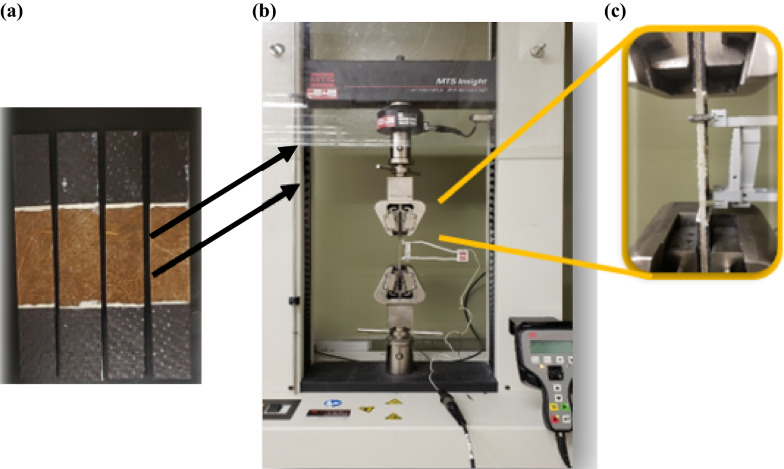
**a** Cattail composite tensile testing coupons; **b** tensile testing sample loaded in MTS using 2-in. extensometer, and **c** fractured sample after testing

Composite test coupons were bonded with tabs to the gripped ends using a room temperature curing adhesive to avoid crushing of the gripped ends during tensile testing. The tabs manufactured using four plies of woven carbon epoxy prepregs were bonded to the composite panels. 127-mm long and 20-mm wide composite test coupons were cut from these panels using a Micro-Matic Precision Wafering Machine (manufactured by Micromech Mfg. Corp.). A slow feed rate of 10 mm/min was used to prevent excessive heat evolution and damage to the edges of test coupons. Edges of the prepared test coupons were ground progressively using 80, 180, 240, 320, and 400 grit silicon carbide papers and polished further using alumina powder to remove any damage due to cutting. Prepared testing coupons, MTS tensile testing machine, and the fractured cattail composite samples after tensile testing are shown in Fig. 2a–c, respectively.

#### Density and fiber volume fraction measurement

The densities of the cattail fibers, the Stypol resin, and the manufactured composites were measured using Helium Pycnometer (Model#UPY-32, UPY-32 T; v-5.04 manufactured by Quantachrome Instruments) according to ASTM D4892-89 (ASTM 2004). The *V*_f_ in composite was calculated using Eq. (3), assuming 100% dense composite:

3 $$V_{{\text{f}}} \left( \% \right) = \frac{{\rho_{{\text{c}}} - \rho_{{\text{m}}} }}{{\rho_{{\text{f}}} - \rho_{{\text{m}}} }} \times 100$$

where *ρ*_f_*, ρ*_m_*, ρ*_c_ are the density of the fiber, the resin, and the composite, respectively.

#### Scanning electron microscopy (SEM) analysis

The fractured surfaces of the composite test coupons from the tensile test were examined in a scanning electron microscope (FEI Quanta 650 FEG ESEM from Thermo Fisher Company, USA) at an accelerating voltage of 10.0 kV. Prior to the SEM analysis, the fractured cattail composite test coupons were coated with a thin layer of gold–palladium film (20 nm) using a Desk II Cold Sputter Etch Unit under the chamber pressure of 30 mTorr.

## Results and discussion

### Fiber extraction

**Fig. 3:**
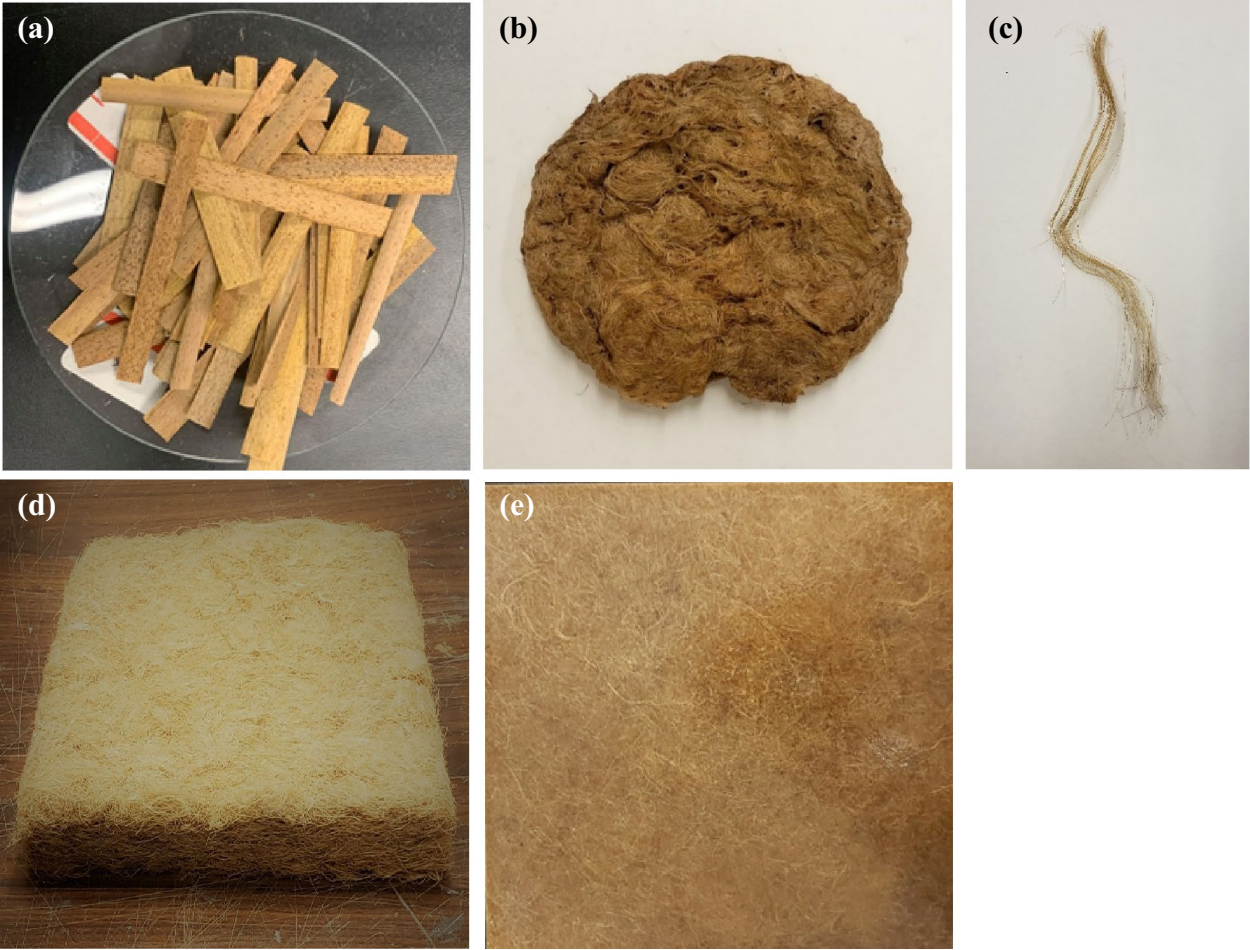
**a** Cattail leaves; **b** extracted and dried fibers; **c** individualized fiber; **d** non-woven cattail mat; **e** non-woven cattail composite

**Fig. 4:**
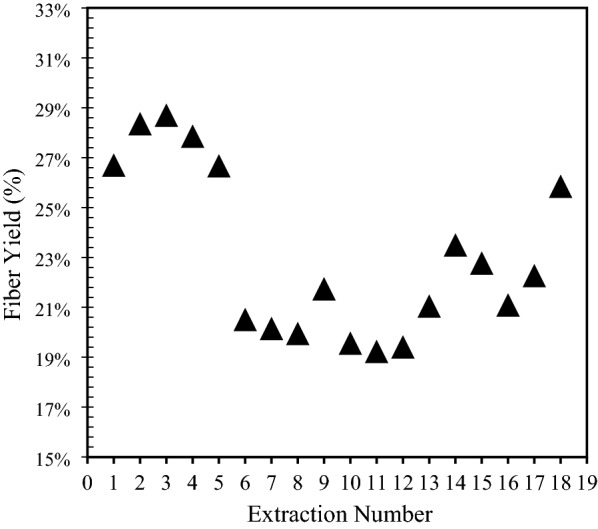
Cattail fiber yield for various extraction runs

The cattail plant leaves, the extracted fibers after drying, and the individualized fibers are shown in Fig. 3. The yield of cattail fiber, extracted in this study using optimum conditions (90 °C for 4 h), varied between 18 and 30% as shown in Fig. 4. The cattail plants were not grown in a controlled environment for this study and collected from wetlands in Winnipeg; therefore, the variation in the measured fiber yield is believed to be due to the difference in cultivar. Also, the fiber yield realized in this study is less than the previously reported cattail fiber yield (40%) (Hasan 2019). This could be due to the use of green cattail plants in the current study in contrast to mature dried plants used by Hasan (2019). Other factors that could have affected the fiber yield are the differences in the types of alkali as well as the extraction time and temperature (Rahman et al, 2021; Shuvo et al. 2020; Sadrmanesh et al. 2019, 2021).

### Physical properties of cattail fibers

**Fig. 5:**
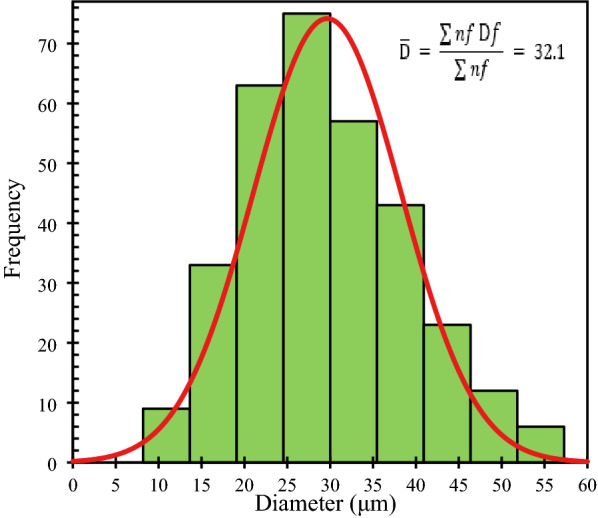
Distribution in diameter of cattail fiber

The length of the extracted cattail fiber depended on the length of cut leaves before extraction. The fiber length after extraction varied between 4 and 12 cm while the diameter varied between 13 and 53 μm exhibiting a normal distribution as shown in Fig. 5. The average fiber diameter is 32.1 μm and the average fiber length is 6.98 cm. While the diameter is smaller, the length is longer than that of flax or hemp fibers as shown in Table 1.

**Table 1:** Physical and mechanical properties of flax, hemp, and cattail fiber

Parameters	Flax	Hemp	Cattail
Length (cm)	6.64 (2.3)	0.4–21^a^	6.98 (1.2)
Diameter (µm)	80.2 (32.7)	138.3 (31.9)^a^	32.1 (8.6)
Density (g/cm^3^)	1.49 (0.004)	1.57 (0.003)	1.39 (0.005)
Tensile strength (MPa)	180.1 (126.1)	172.1^a^	172.3 (99.3)
Modulus (GPa)	11.3 (10.7)	28.5^a^	18.1 (9.7)

^a^Fahimian 2013

The experimental density values for cattail fiber and Stypol resin are 1.39 g/cm^3^ (SD = 0.005) and 1.16 g/cm^3^ (SD = 0.001), respectively. The cattail fiber is lighter than flax and hemp fibers (Table 1), which is believed to be due to the hollow structure of the cattail fiber (Rahman et al. 2021). The recorded density value for Stypol resin in this study (1.16 g/cm^3^) is lower than the previously reported density value (1.3 g/cm^3^) for Stypol resin (Fahimian 2013). This could be due to the differences in the amount of initiator and resin batches used in these two studies. The density of cattail fiber extracted in this study (1.39 g/cm^3^) is higher than the density value of 1.26 g/cm^3^ reported by Mortazavi and Moghadam (2009). This difference could be due to the use of two different density measurement techniques; Mortazavi and Moghadam (2009) used liquid media while the current study used helium gas. Such difference has been reported by other researchers for flax fibers. The density of flax fiber is varied with measurement method—2.48 g/cm^3^ in water, 2.55 g/cm^3^ using linear density method and 1.5 g/cm^3^ using helium pycnometry method (Truong et al. 2009).

**Fig. 6:**
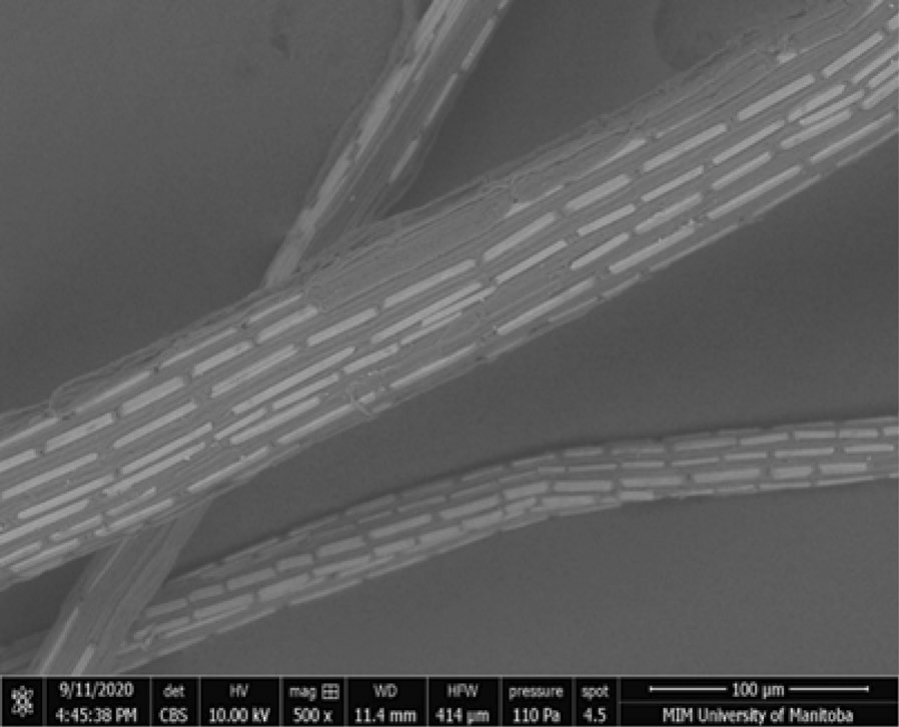
SEM image of cattail fiber

An SEM image of cattail fiber is shown in Fig. 6. The rectangular calcium oxalate plates and pit areas (without oxalate plates) can be seen on the surface of the virgin cattail fibers. These plates lie along the fiber axis and their length and width vary from location to location.

### Mechanical properties of cattail fiber

**Fig. 7:**
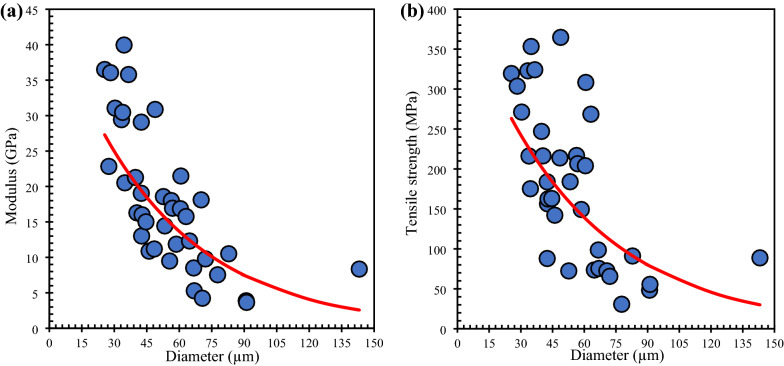
Variation in **a** tensile modulus and **b** tensile strength of virgin cattail with fiber diameter

The experimental tensile modulus (*E*_f_) and tensile strength (*σ*_f_) of virgin cattail fiber varied with fiber diameter as shown in Fig. 7a and b, respectively. The tensile modulus (*E*_f_) decreased with an increase in fiber diameter (*D*_f_). A similar trend was observed for tensile strength. This relationship is modeled by the empirical equation given in Eqs. (4) and (5). This trend is similar to that observed in flax fibers (Shadhin et al. 2021) and hemp fibers (Fahimian 2013). The tensile strength varied from 9 to 365 MPa (avg. = 172.3 ± 99.3) and tensile modulus varied from 3 to 40 GPa (avg. = 19.1 ± 9.6). Similar variations in the tensile strength and the modulus have been reported for other bast fibers (Li et al. 2007; Joffea et al. 2003; Ali 2013; Ibrahim et al. 2018). The modulus and the strength of cattail fibers have been found to change with moisture content with a maximum after conditioning at 75.5% RH for 72 h during which the fibers absorbed ~ 15% moisture (Shadhin 2021). The fibers used in this study were stored in lab atmosphere with a relative humidity of ~ 55%. It can be inferred from Table 1 that the tensile modulus of cattail fibers is higher than flax fibers but lower than that of hemp fibers. The tensile strength of cattail fibers is comparable to that of hemp and flax fibers. While its specific strength (strength/density) is higher than that of hemp and flax fibers, its specific modulus (modulus/density) is in between that of hemp and flax fibers. These results suggest that the mass and the mechanical properties of composites manufactured with cattail fibers could be similar to those of hemp fiber composites:

4 $$E_{{\text{f}}} = 45.57\,\exp \,\left[ { - 0.02\,\left( {D_{{\text{f}}} } \right)} \right]$$

5 $$\sigma_{{\text{f}}} = 422.03\,\exp \,\left[ { - 0.018\,\left( {D_{{\text{f}}} } \right)} \right]$$

### Mat characterization

#### Physical properties of non-woven cattail mat

The areal density, the mat thickness, the permeability, and the *V*_f_ of non-woven mats used in the manufacturing of composites at three consolidation pressures are tabulated in Table 2. The three mats were manufactured using three batches of extracted fibers. Due to the marginal difference in the fiber yield among the three batches, the areal density and the thickness of the three manufactured mats varied marginally as observed in Table 2. Despite using the same weight to compress the fibers during the manufacturing of the mat, the thickness and hence the *V*_f_ in the mat varied from one mat to another. These variations are due to variation in the areal density as well as due to heterogeneous distribution of fibers within the non-woven mat. Shadhin (2021) manufactured flax and flax–hemp hybrid non-woven mat with similar areal density following the same procedure. Under the same weight (3 kg) used to compress the fibers during mat preparation cattail mat achieved less compaction indicated by higher thickness (19–21 mm) than flax (16.3 mm) and flax–hemp mat (13.9 mm) (Shadhin 2021). This is believed to be due to the greater length of cattail fibers than flax and hemp (Table 1).

**Table 2:** Physical properties of non-woven cattail mat used in manufacturing composites using three consolidation pressures

Consolidation pressure (kPa)	Areal density of mat (g/m^2^), SD^a^	Mat thickness before consolidation (mm), SD^a^	Fiber volume fraction in mat, *V*_f_ %, SD^a^	Out-of-plane permeability × 10^–11^ (m^2^), SD^a^
101	845	19.3 (0.3)	3.2 (0.06)	5.9 (0.03)
260	921	17 (0.2)	3.9 (0.1)	4.7 (0.2)
560	974	21 (0.2)	3.3 (0.1)	4.9 (0.3)

^a^SD—standard deviation, *N* = 3

#### Mat permeability

The out-of-plane permeability of each cattail mat was measured at three different locations and the experimental values are tabulated in Table 2. The mean experimental out-of-plane permeability of cattail mats varied from 4.38 × 10^–11^ to 5.97 × 10^–11^ m^2^. The increase in permeability with the decrease in *V*_f_ (alternatively increase in % porosity) is evident.

The out-of-plane permeability of cattail non-woven mats in Table 2 is higher than that of the non-woven flax (2.5 × 10^–11^ m^2^) and hemp (2.8 × 10^–11^ m^2^) mats measured using the air medium (Shadhin 2021). The higher permeability values of the cattail mat when compared to those of flax and hemp mats are due to the lower *V*_f_ of the cattail mat despite similar areal density. This is believed to be due to longer cattail fibers, which would have resulted in a lower level of compaction than hemp or flax fibers during mat manufacturing.

### Composite characterization

#### Effect of consolidation pressure on structure of composite

The measured values for the thickness, the density, and *V*_f_ of cattail composites manufactured at various consolidation pressures, applied during manufacturing, are tabulated in Table 3. The decrease in the thickness due to consolidation was maximum at the VARTM pressure of 101 kPa (68.6%). Subsequent consolidation decreased with the increase in pressure; 54.3% when the pressure was increased from 101 to 260 kPa and 18.9% when the pressure was increased from 260 to 560 kPa, as observed in Table 3.

**Table 3:** Thickness, density, and fiber volume fraction of cattail composites manufactured at various consolidation pressures

Consolidation pressure (kPa)	Composite thickness after curing (mm), SD^a^	Density of composite (g/cm^3^), SD^a^	Fiber volume fraction in composite, *V*_f_ %, SD^a^
101	6 (0.8)	1.19 (0.003)	11.2 (0.4)
260	2.7 (0.04)	1.23 (0.005)	30.4 (0.6)
560	2.2 (0.04)	1.22 (0.002)	26.1 (0.4)

^a^SD—standard deviation, *N* = 5

The *V*_f_ in the cattail composites increased with the increase in the consolidation pressure to a maximum value at 260 kPa. Instead of increasing further, it marginally decreased when the consolidation pressure was increased further to 560 kPa. This is believed to be due to the difference in the compaction behavior of the mats owing to differences in the areal density and the thickness of three mats (Table 2). Since the fibers were dropped into the mold manually during the manufacturing of the non-woven mat, their arrangement or packing within the three mats could also have been different resulting in the observed anomaly in consolidation when the pressure was increased to 560 kPa.

#### Mechanical properties

**Fig. 8:**
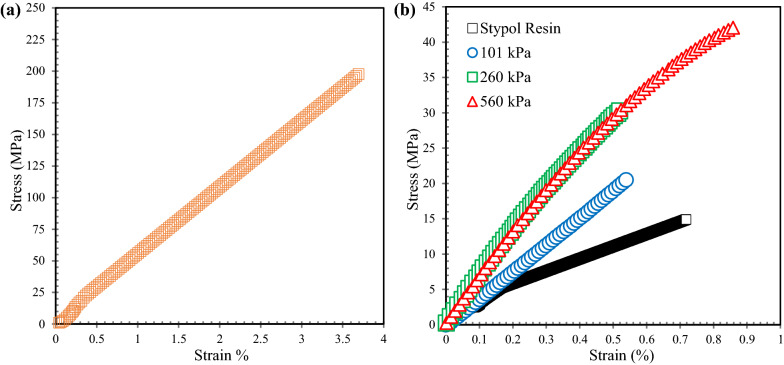
Representative stress–strain curve for **a** cattail fiber and **b** Stypol resin and cattail mat composites manufactured at different pressures

The tensile stress–strain curves for the cattail fiber-reinforced composites, manufactured at different molding pressures, along with those of the Stypol resin are plotted in Fig. 8b. Also, a representative tensile stress–strain curve for the virgin cattail fiber is shown in Fig. 8a. It can be inferred that the cattail fiber reinforces the neat resin significantly; however, the level of reinforcement varied with the manufacturing pressures due to variation in *V*_f_ with consolidation pressure. The modulus, tensile strength, and failure strain of composites, obtained from these plots, are tabulated in Table 4.

**Table 4:** Mechanical properties of cattail fiber-reinforced composite

Mat content	Consolidation pressure (kPa)	Longitudinal modulus (GPa) SD^a^	Tensile strength (MPa), SD^a^	Strain at break (%), SD^a^
100% cattail	101	4.6 (0.6)	18.6 (3.2)	0.4 (0.1)
100% cattail	260	7.0 (0.2)	34.0 (3.8)	0.5 (0.1)
100% cattail	560	6.5 (0.2)	44.1 (2.7)	1.0 (0.1)

^a^SD—standard deviation, *N* = 5

**Fig. 9:**
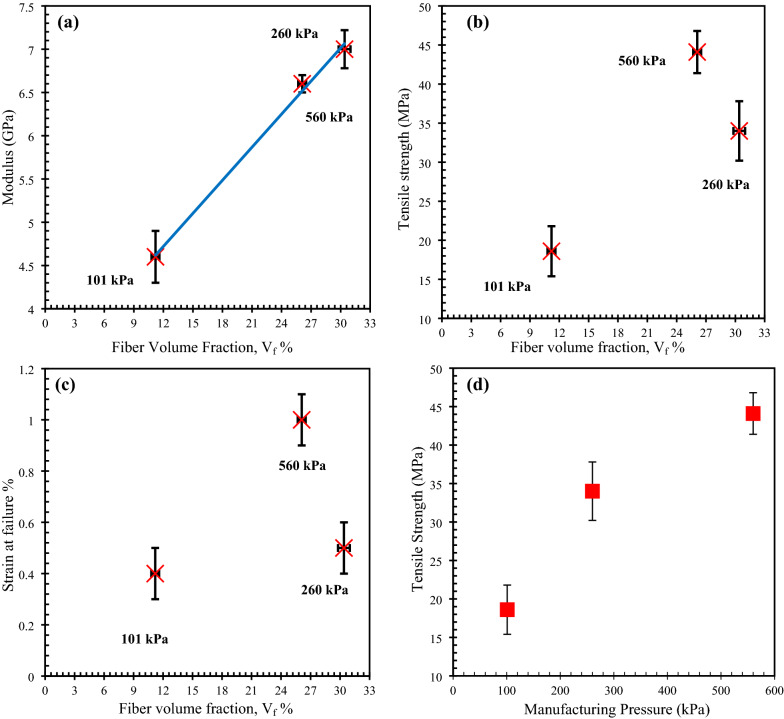
Relationship between **a** tensile modulus and *V*_f_; **b** tensile strength and *V*_f_; **c** strain at failure, and *V*_f_; **d** tensile strength and consolidation pressure—for cattail composites

It can be inferred from this table that the Stypol resin is significantly reinforced by the cattail fibers. The magnitude of reinforcement depends on the consolidation pressure due to the change in the *V*_f_ of composite with consolidation pressure. The tensile modulus, the strength, and the failure strain of cattail composites are plotted as a function of *V*_f_ in Fig. 9a–c, respectively.

The tensile modulus increased with pressure until 260 kPa, beyond which it decreased when the pressure was increased further to 560 kPa. This is due to an increase in *V*_f_ from 3.2 to 3.9% in the mat to 11.2% at 101 kPa, which increased to 30.4% at 260 kPa before decreasing to 26.1% at 560 kPa. The reason for this trend in *V*_f_ is due to difference in the consolidation of the mats, as discussed in the previous section. The linear relation between the modulus and the *V*_f_ in Fig. 9a clearly establishes the effect of consolidation pressure in increasing the *V*_f_ and the modulus of the cattail composite. The increase in the modulus and the strength was found to be statistically significant in two-tailed *T*-tests while comparing the values among different groups at different consolidation pressures (Shadhin 2021).

**Fig. 10:**
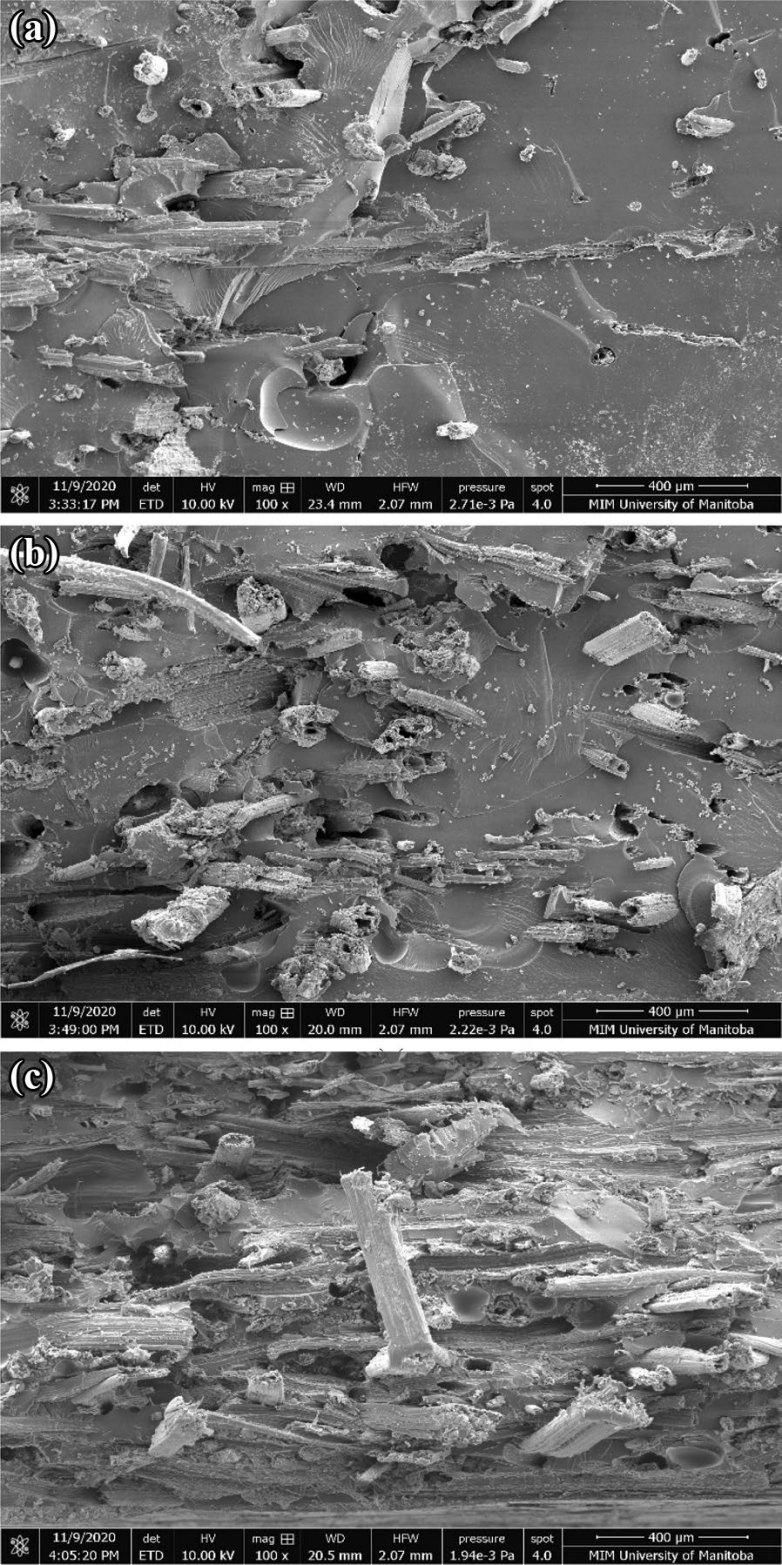
SEM of fractured surface of cattail composite at: **a** 101 kPa, **b** 260 kPa, and **c** 560 kPa

The tensile strength increased with the increase in the consolidation pressure (until 260 kPa) during which the *V*_f_ also increased. When the pressure was increased further to 560 kPa, the strength increased further from 34 MPa (± 3.8) at 260 kPa to 44.1 MPa (± 2.7), despite lower *V*_f_ at 560 kPa as shown in Fig. 9b and d. A similar trend in the fracture strain is observed in Fig. 9c. Typically, the failure strain would decrease with the increase in the tensile strength. Lower failure strain at 101 kPa and 260 kPa when compared to that at 560 kPa, despite lower strength suggests that premature failure, perhaps due to stress concentration, in test specimens manufactured at 101 kPa and 260 kPa could be the reason for the lack of trend in the strength and the failure strain with *V*_f_. The relatively highly rough (i.e., ductile) fracture surface of resin area in Fig. 10c for 560 kPa when compared to smooth (i.e., brittle) fracture surface of resin area in Fig. 10a and b for 101 kPa and 260 kPa, appears to confirm this interpretation.

Although the cattail composite exhibited a similar level of reinforcement indicated by the superimposed stress–strain curve in Fig. 8b at 260 and 560 kPa, the mechanical properties of the cured composite varied with fiber characteristics (fiber length, fiber orientation) and non-woven mat properties (areal density, *V*_f_). Mats with higher starting areal density and shorter fiber length would achieve higher consolidation at higher pressures resulting in higher mechanical properties of the composite. However, the maximum value for the areal density of the non-woven cattail mat would be limited by the permeability required for successful consolidation while manufacturing composite, since permeability is inversely related to areal density (i.e., *V*_f_). At VARTM pressure, the *V*_f_, the modulus, and the tensile strength of cattail composites are similar in magnitude to those of flax and hemp fiber composites (Shadhin 2021), demonstrating the suitability of cattail fibers as reinforcements in composites.

## Conclusions

The cattail fibers, extracted using alkali retting, exhibited a normal distribution in diameter; the modulus and the strength varied inversely with the diameter, similar to flax and hemp fibers. The non-woven mats manufactured using these fibers were used with unsaturated polyester and VARTM to manufacture composites. The out-of-plane permeability of non-woven cattail mats was higher than those of flax or hemp mats with similar *V*_f_. The mechanical properties of cattail fiber and its composites are comparable to the published properties of hemp/flax fibers and their composites, demonstrating the suitability of cattail fibers as reinforcement. Future research should consider modification of the cattail fiber surface to enhance the bonding with the resin matrix of composites as well as study the effect of needle punching on the properties of non-woven mats and composites manufactured using these mats.

## Data Availability

All data are fully available without restriction.

## Abbreviations

VARTM: Vacuum-assisted resin transfer molding
PMC: Polymer matrix composite
NFRC: Natural fiber-reinforced composites
BFs: Biomass fibers
WBFs: Waste biomass fibers
ASTM: American Society for Testing and Materials
GSM: Gram per square meter
SEM: Scanning electron microscopy
